# Supplementary material for: Plasmalogens: Free Radical Reactivity and Identification of Trans Isomers Relevant to Biological Membranes
Source: Biomolecules. 2023 Apr 24;13(5):730. doi: 10.3390/biom13050730 (PMC10216421; doi:10.3390/biom13050730)
Supplement: Supplementary file 1 [file biomolecules-13-00730-s001.zip › biomolecules-2319973-supplementary.pdf]

Plasmalogens: Free Radical Reactivity and Identification of Trans Isomers Relevant to Biological Membranes

Carla Ferreri 1,*, Alessandra Ferocino 1,†, Gessica Batani 1, Chryssostomos Chatgilialoglu 1,2, Vanda Randi 3, Maria Vittoria Riontino 3, Fabrizio Vetica 1,‡ and Anna Sansone 1

SUPPORTING INFO

Table S1: FAME yield (%) derived from the transesterification of C18plasm20:4-PC (0.32 mmol/mL) under three different conditions: A) basic condition 0.5M KOH/MeOH at room temperature (r.t.); B) acidic condition 1: 1.5% HCl/MeOH at 100 °C; C) acidic condition 2: 14% BF_3/MeOH at 50 °C, as described in the Experimental Part of the main text.

Fatty acid derivative [a]	Transesterification conditions		
	Condition A µmol/mL; yield(%) (*n*=3)	Condition B µmol/mL; yield (%) (*n*=3)	Condition C µmol/mL; yield (%) (*n*=3)
18:0 DMA	n.d.	0.16[#]; 52%	0.095[#]; 32.5% [£££]
20:4 ARA Methyl ester	0.32[#]; 100%	0.29[#]; 92%**	0.18[#]; 56%[###;£££]

[a]cis FAME and DMA identified by standard references, quantified using heptadecanoic acid (17:0) as internal GC standard, as described in Methods. [#]yields of the three repetitions were found with errors <0.005%. Statistics: unpaired *t* test: **A vs B; p value ≤ 0.005; [###]A vs C ; p value ≤ 0.0001; [£££]B vs C; p value ≤ 0.0001. n.d = not detected

Table S2. Transformation of linoleic acid (9cis,12cis-18:2) residues of soybean lecithin into fatty acid methyl ester (FAME) under alkaline (0.5 M KOH/MeOH at r.t.) and acidic (1.5% HCl/ MeOH at 100 °C) conditions.

FAME [1]	Alkaline condition yield % Mean ± sd (*n*=3)	Acidic condition yield % Mean ± sd (*n*=3)
18:2	100 ± 0	93 ±3

[1] FAME identified by standard reference, quantified using heptadecanoic acid (17:0) as internal GC standard, as described in Methods.

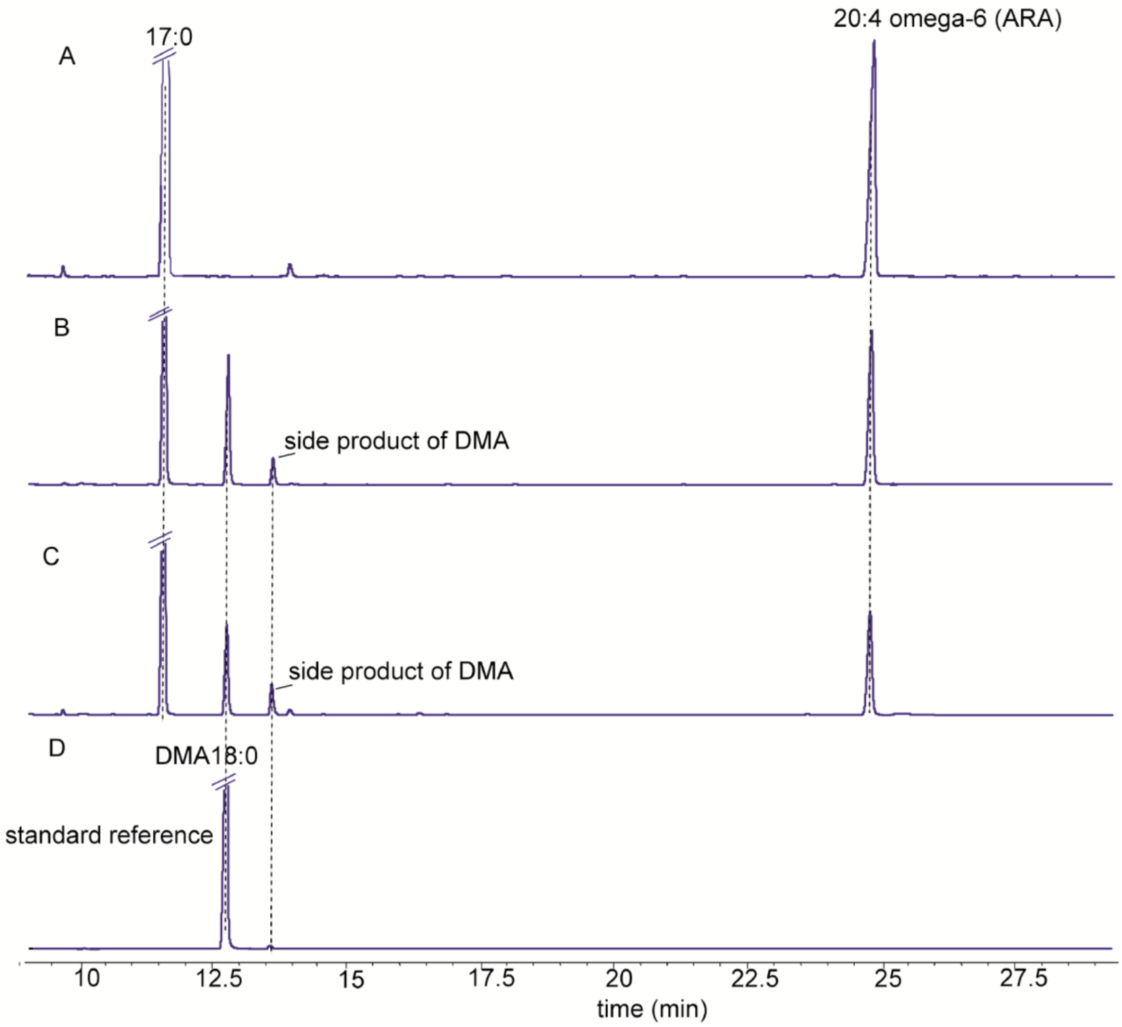

Figure S1: GC chromatograms of plasmalogen (C18 plasm 20:4-PC) transesterification performed in three conditions: A (alkaline: 0.5 M KOH/MeOH, r.t.); B (acidic: 1.5% HCl/MeOH, 100 °C) ; C (acidic: 14% BF_3/MeOH, 50 °C); In trace D the standard reference of 18:0 DMA is shown, together with DMA side product.

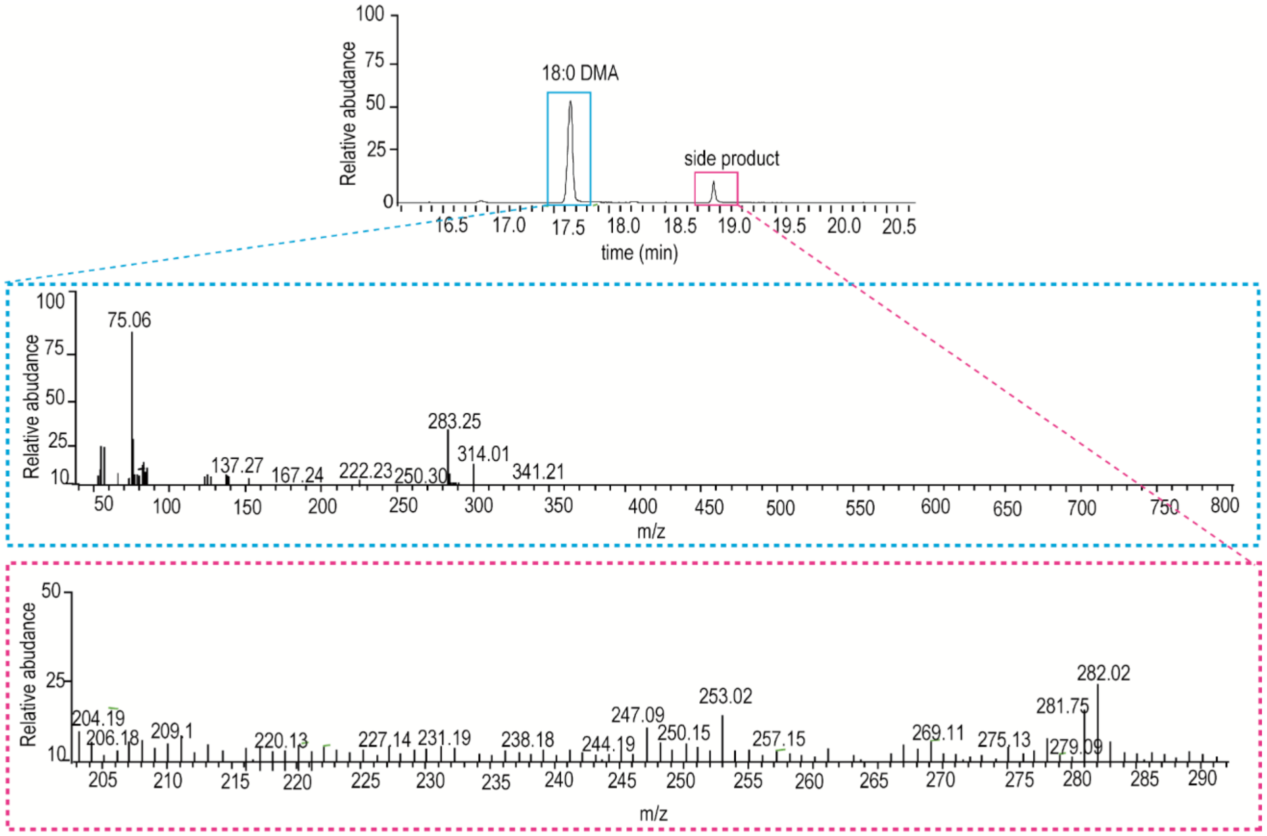

Figure S2: GC/MS chromatogram showing 18:0 DMA and the side product after plasmalogen transesterification in acidic condition; in the cyan box diagnostic fragments corresponding to 18:0 DMA with molecular peak (*m/z* 283.75); in the purple box fragmentation of the 18:0 DMA by-product.

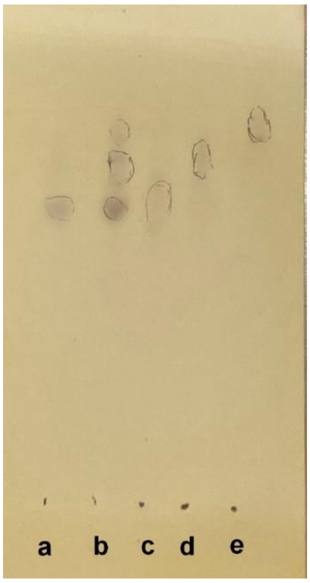

Figures S3. Ag-TLC monitoring of the photoisomerization (b) of plasmalogen (C18 plasm 20:4-PC) (a) (eluent $CHCl_3$:MeOH:H_20 = 4.5:2.3:0.2) showing separation of the fractions (c,d,e). The (d) and (e) fractions correspondto trans-containing plasmalogens and attempts for their isolation were made by the same eluent using preparative Ag-TLC. Fractions were not stable during isolation, and it was not possible to characterize them further.

C18 plasm 20:4-PC - Plasmalogen (starting material)

^{1}H NMR (500 MHz, Benzene-d6) δ 6.22 (bs, 1H, -OC*H*=C alkenyl ether), 5.62 (m, 1H, CH glycerol), 5.52 (m, 8H, alkenyl H), 4.55 (q, J = 10.0 Hz, 1H, CH alkenyl ether), 4.45 (bs, choline CH_2), 4.30 (m, 2H, glycerol CH_2), 4.25-4.16 (m, 2H, glycerol CH_2), 3.84 (bm, 2H, choline H), 3.49 (s, 9H, $N(CH_3)_3$) 2.92 (m, 6H, bisallylic CH_2), 2.42 (bs, 2H, CH_2 alpha to C=O), 2.34 (q, J = 10 Hz, C*H*$_2$C=CH allylic), 2.18 (m, 2H, C*H*$_2$CH=CHO), 2.09 (q, J = 10 Hz, C*H*$_2$C=CH allylic), 1.83, (q, J=10Hz, 2H, CH_2*CH*$_2$$CH_2$C(O)OR), 1.52-1.27 (m, 34H, CH_2), 0.91 (t, J = 10 Hz, 6H, 2x CH_3).

.

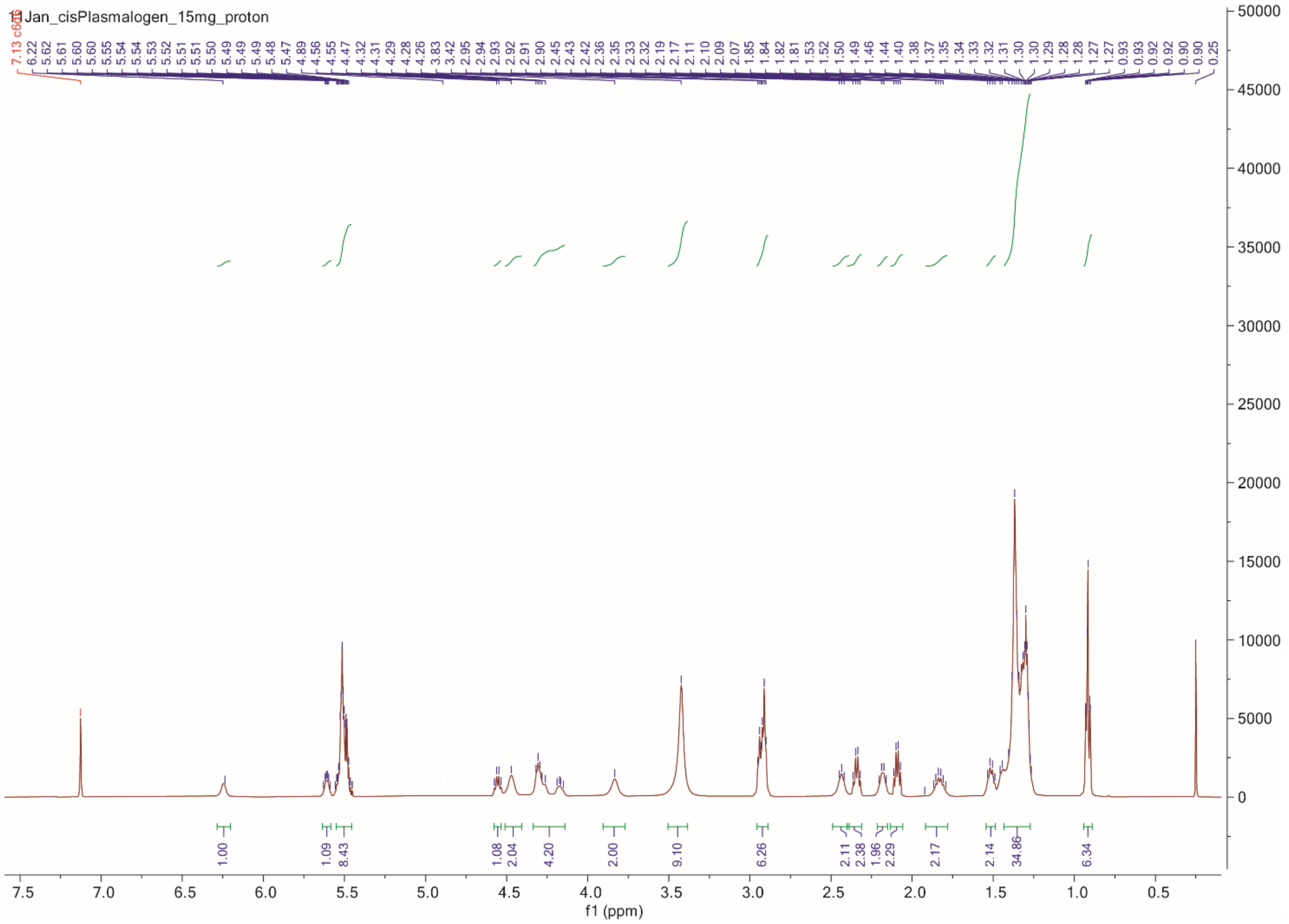

Figure S4. ^{1}H NMR spectrum of commercially available plasmalogen in C_6D_6

C18 plasm 20:4-PC - Plasmalogen (starting material)

^{13}C NMR (126 MHz, Benzene-d6) δ 172.84, 145.66, 130.33, 129.27, 128.77, 128.62, 128.31, 128.30, 127.99, 127.47, 107.07, 72.50, 72.44, 71.20, 66.06, 63.60, 59.59, 53.98, 33.87, 32.03, 31.54, 30.26, 30.09, 30.07, 30.05, 30.02, 30.00, 29.96,29.33, 29.85, 29.81, 29.53, 29.43, 27.29, 26.60, 25.77, 25.73, 25.71, 25.11, 24.41, 22.80, 22.67, 14.04, 14.02.

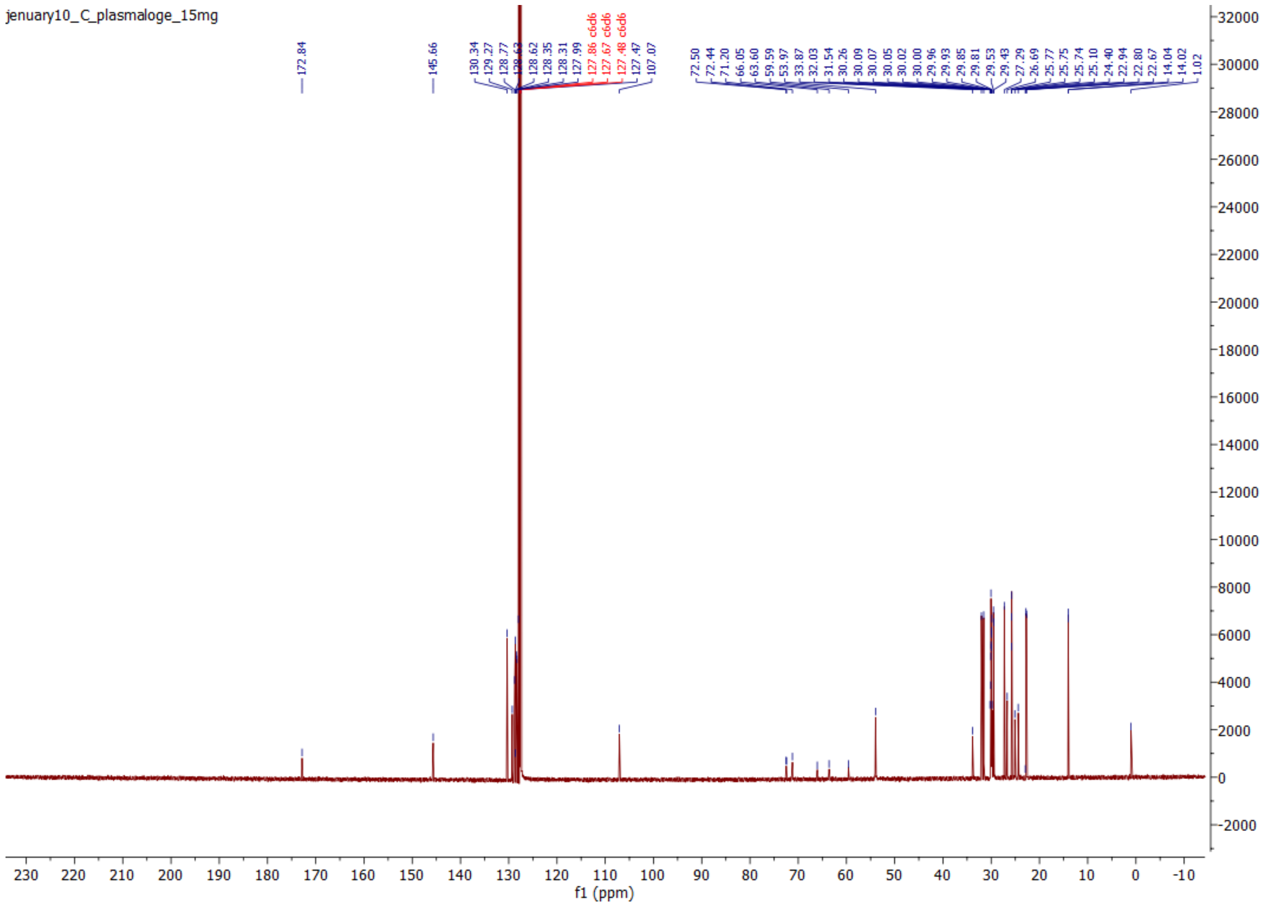

Figure S5. ^{13}C NMR spectrum of commercially available plasmalogen in C_6D_6

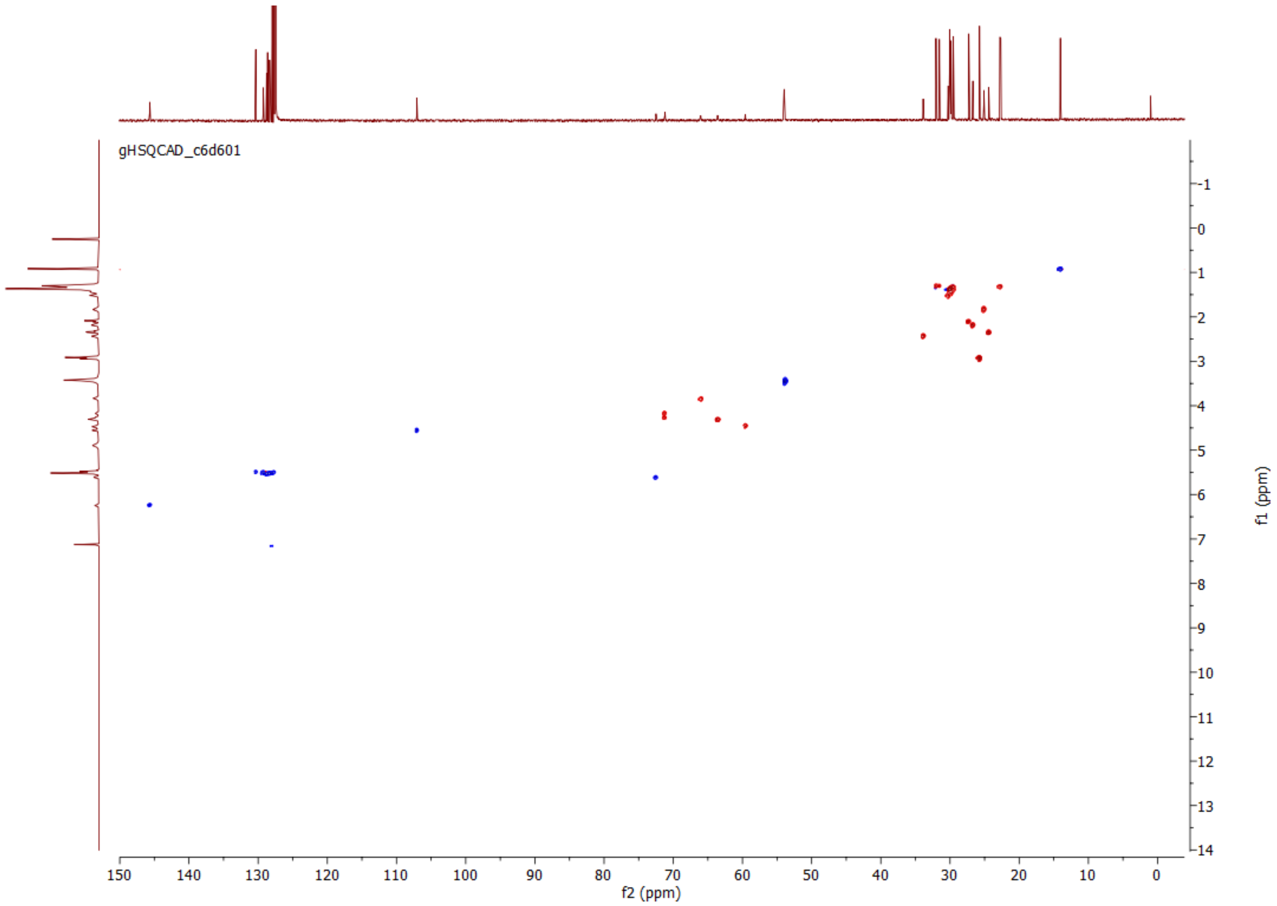

Figure S6a: HSQC 2D-NMR spectrum of commercially available plasmalogen in C_6D_6

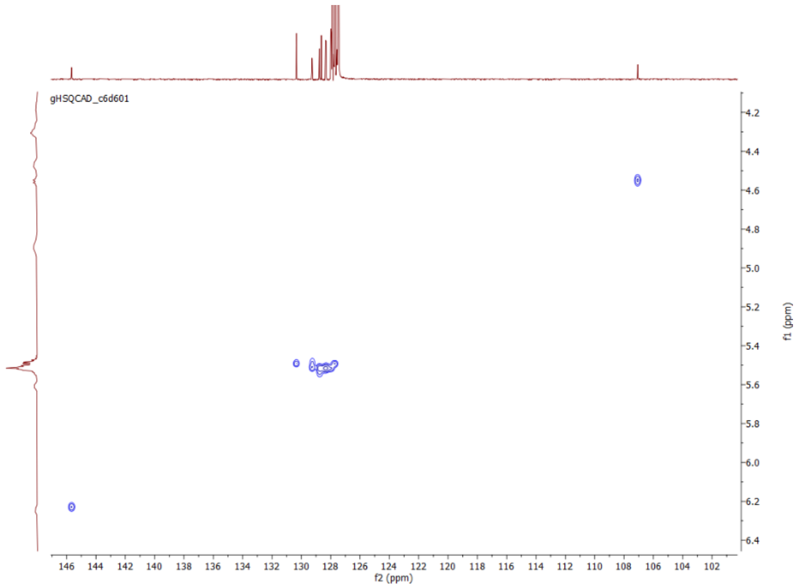

Figure S6b: HSQC 2DNMR 4.1-6.4 ppm region containing the resonances of the vinyl ether and alkenyl moieties of all cis plasmalogen

Photoisomerization mixture containing cis/trans C18 plasm 20:4-PC

^{1}H NMR (500 MHz, Benzene-d6) δ **6.37** (d, J=10Hz, 28% trans -OC*H*=C alkenyl ether), 6.18 (bs, 1H, cis -OC*H*=C alkenyl ether), 5.62 (m, 1H, CH glycerol), 5.52 (m, 8H, alkenyl H), **4.92** (m, 1H, trans CH alkenyl ether) 4.50 (q, J = 10.0 Hz, 1H, cis CH alkenyl ether), 4.45 (bs, choline CH_2), 4.30 (m, 2H, glycerol CH_2), 4.25-4.16 (m, 2H, glycerol CH_2), 3.84 (bm, 2H, choline H), 3.49 (s, 9H, $N(CH_3)_3$) 2.92 (m, 6H, bisallylic CH_2), **2.78** (m, trans bisallylic H), 2.42 (bs, 2H, CH_2 alpha to C=O), 2.34 (q, J = 10 Hz, CH_2C=CH allylic), 2.18 (m, 2H, CH_{62}CH=CHO), 2.09 (q, J = 10 Hz, CH_2C=CH allylic), **1.97** (m, trans CH allyl), 1.98-2.04 (m, 1.83 (q, J=10Hz, 2H, CH_2C$H_2$$CH_2$C(O)OR), 1.52-1.27 (m, 34H, CH_2), 0.91 (t, J = 10 Hz, 6H, 2x CH_3).

See enlargements in the main text – Figure 2

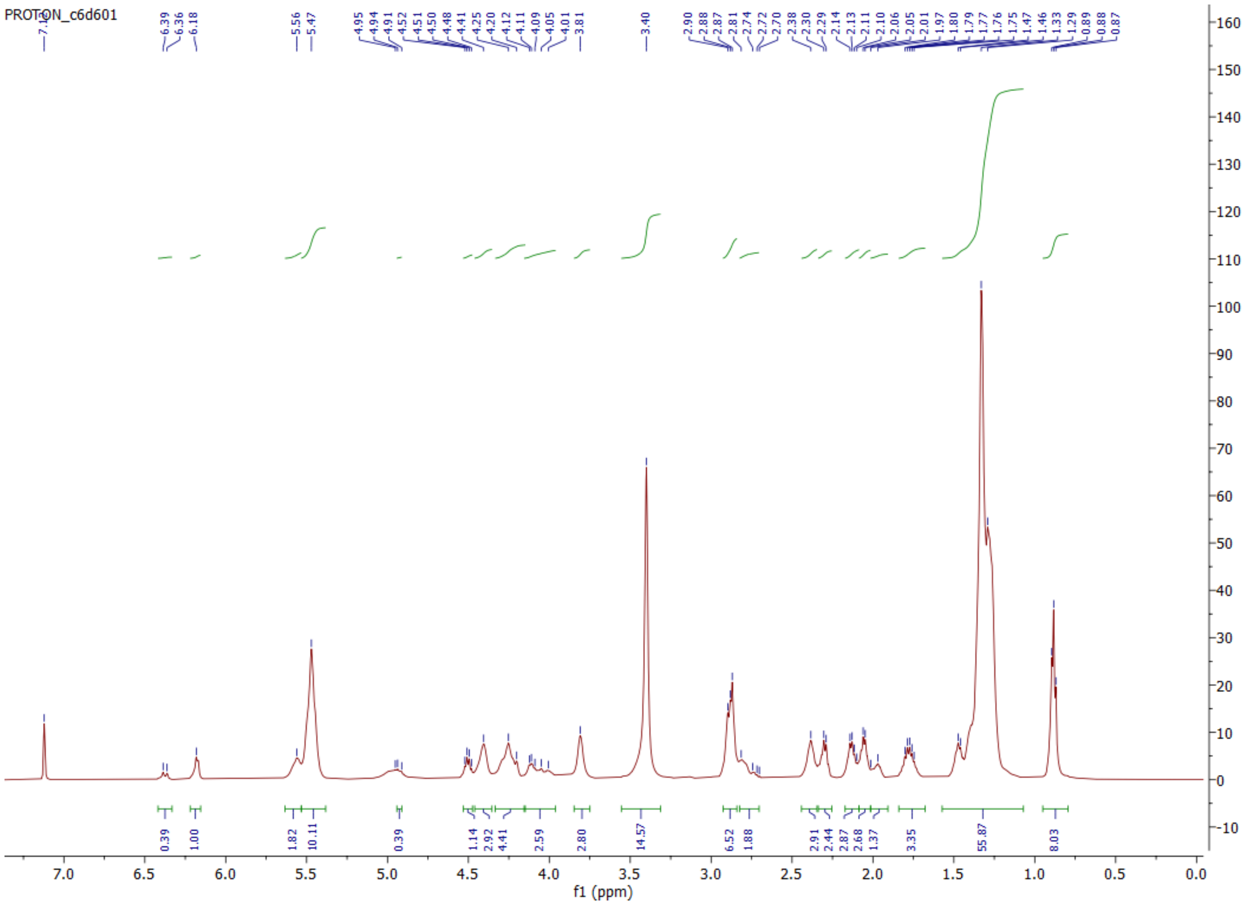

Figure S7. ^{1}H NMR spectrum in C_6D_6 of the reaction mix of plasmalogen isomerization after 4 min photolysis under the condition described in the Methods

Photoisomerization mixture containing cis/trans C18 plasm 20:4-PC

^{13}C NMR (126 MHz, Benzene-d6) δ 172.84, **146.32**, 145.66, **130.90**, **130.51**, 130.34, **130.26**, **130.21**, 129.25, 128.76, 128.62, 128.33, 128.30, 127.98, 127.94, 107.07, **104.65**, 72.50, 72.44, 71.20, 66.06, 63.60, 59.59, 53.98, 33.87, 32.03, 31.54, 30.26, 30.09, 30.07, 30.05, 30.02, 30.00, 29.96,29.33, 29.85, 29.81, 29.53, 29.43, 27.29, 26.60, 25.77, 25.73, 25.71, 25.11, 24.41, 22.80, 22.67, 14.04, 14.02.

See enlargements in the main text – Figure 2

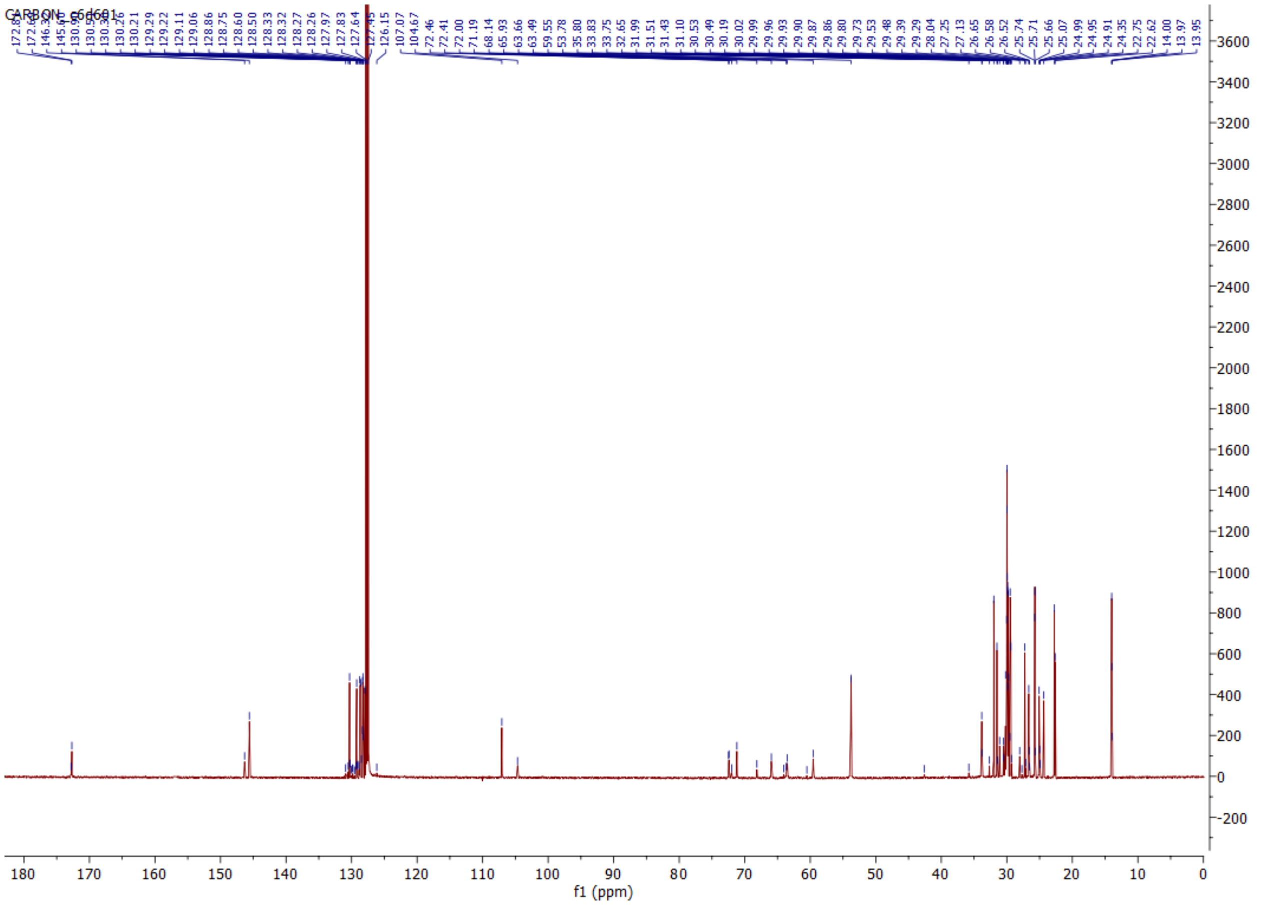

Figure S8. ^{13}C NMR spectrum in C_6D_6 of the reaction mix of plasmalogen isomerization after 4 min photolysis under the condition described in the Methods.

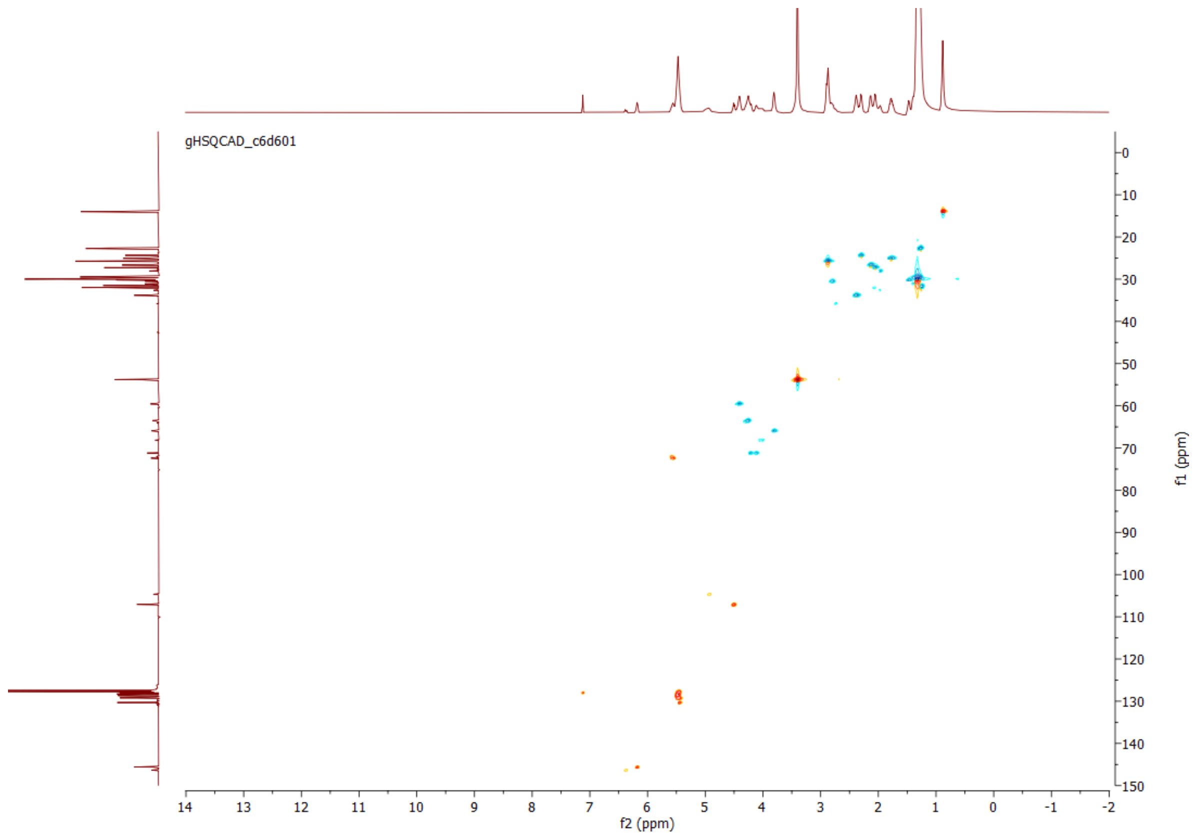

Figure S9a: HSQC 2D NMR spectrum of the mixture cis-trans plasmalogen in C_6D_6

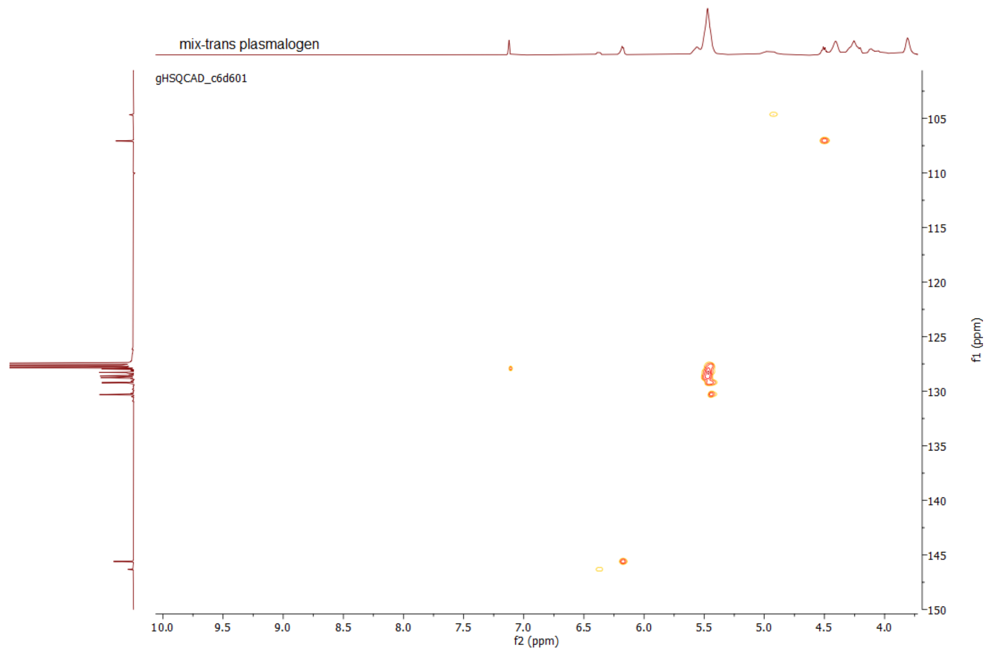

Figure S9b: HSQC 2D NMR 3.9-10 ppm enlargment containing the vinyl ether and alkenyl resonances in the mixture of cis-trans plasmalogen

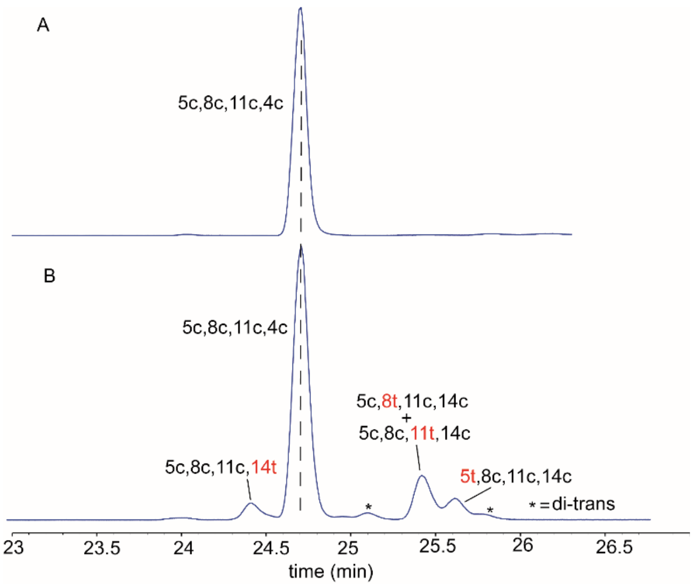

Figure S10: Enlargement of GC traces referred to: A, standard reference of arachidonic acid methyl ester (Ara-Me); B, mixture of cis and monotrans-Ara-Me obtained by transesterification of the plasmalogen photolysis mixture after 4 min of irradiation (traces of di-trans Ara-Me (*) are also detected).

SAPC Liposome Incubation

See Methods for details

Table S3. SAPC liposome (1 mM) aqueous suspension under oxidation condition incubated at 37 °C in open air for 15 h. Experiments were performed in triplicates.

**OXIDATION CONDITION**	Ara loss n=3
$Fe(NH_4)_2(SO_4)_2 \times 6H_2O$ (10 μM) H_2O_2(100 μM)	73.2±0.7

Details of Figure 3

Table S4. Follow-up of the isomerization of C18 plasm 20:4-PC (0.125 mM) in presence of 2-mercaptoethanol (0.5 equiv) at different times (1,2.5, 4 min): **vinyl ether** obtained by NMR in deuterated benzene of the reaction crude; **monotrans Ara** analysed by GC after work-up to obtain arachidonic acid methyl ester (AraMe), as described in Methods. Data are in the main text as Figure 3.

	**vinyl ether** *		**monotrans Ara**§		
Photolysis (min)	trans OC***H***CH	trans OCHC***H***	**5t**,8c,11c,14c	5c,**8t**,11c,14c+5c,8c,**11t**,14c	5c,8c,11c,**14t**
1	12%	11%	3.0%	6.5%	3.1%
2.5	23%	22%	4.6%	10.5%	4.7%
4	33%	32%	7.8%	15.5%	7.5%

*The percentage of isomerization of vinyl ether was obtained by ^{1}H NMR using TMS as reference (see Methods); §the percentages of Ara monotrans isomers were obtained by GC analysis.
